# White Matter Integrity and Nicotine Dependence: Evaluating Vertical and Horizontal Pleiotropy

**DOI:** 10.3389/fnins.2021.738037

**Published:** 2021-10-14

**Authors:** Zhenyao Ye, Chen Mo, Song Liu, Kathryn S. Hatch, Si Gao, Yizhou Ma, L. Elliot Hong, Paul M. Thompson, Neda Jahanshad, Ashley Acheson, Hugh Garavan, Li Shen, Thomas E. Nichols, Peter Kochunov, Shuo Chen, Tianzhou Ma

**Affiliations:** ^1^Maryland Psychiatric Research Center, Department of Psychiatry, School of Medicine, University of Maryland, Baltimore, Baltimore, MD, United States; ^2^Division of Biostatistics and Bioinformatics, Department of Epidemiology and Public Health, School of Medicine, University of Maryland, Baltimore, Baltimore, MD, United States; ^3^School of Computer Science and Technology, Qilu University of Technology, Shandong Academy of Sciences, Jinan, China; ^4^Imaging Genetics Center, Mark and Mary Stevens Neuroimaging and Informatics Institute, Keck School of Medicine of USC, University of Southern California, Los Angeles, CA, United States; ^5^Department of Psychiatry and Behavioral Sciences, University of Arkansas for Medical Sciences, Little Rock, AR, United States; ^6^Department of Psychiatry, The University of Vermont, Burlington, VT, United States; ^7^Department of Biostatistics, Perelman School of Medicine, University of Pennsylvania, Philadelphia, PA, United States; ^8^Oxford Big Data Institute, Li Ka Shing Centre for Health Information and Discovery, Nuffield Department of Population Health, University of Oxford, Oxford, United Kingdom; ^9^Department of Epidemiology and Biostatistics, School of Public Health, University of Maryland, College Park, College Park, MD, United States

**Keywords:** nicotine, white matter integrity, pleiotropy, mediation analysis, causal inference

## Abstract

Tobacco smoking is an addictive behavior that supports nicotine dependence and is an independent risk factor for cancer and other illnesses. Its neurogenetic mechanisms are not fully understood but may act through alterations in the cerebral white matter (WM). We hypothesized that the vertical pleiotropic pathways, where genetic variants influence a trait that in turn influences another trait, link genetic factors, integrity of cerebral WM, and nicotine addiction. We tested this hypothesis using individual genetic factors, WM integrity measured by fractional anisotropy (FA), and nicotine dependence-related smoking phenotypes, including smoking status (SS) and cigarettes per day (CPDs), in a large epidemiological sample collected by the UK Biobank. We performed a genome-wide association study (GWAS) to identify previously reported loci associated with smoking behavior. Smoking was found to be associated with reduced WM integrity in multiple brain regions. We then evaluated two competing vertical pathways: Genes → WM integrity → Smoking versus Genes → Smoking → WM integrity and a horizontal pleiotropy pathway where genetic factors independently affect both smoking and WM integrity. The causal pathway analysis identified 272 pleiotropic single-nucleotide polymorphisms (SNPs) whose effects on SS were mediated by FA, as well as 22 pleiotropic SNPs whose effects on FA were mediated by CPD. These SNPs were mainly located in important susceptibility genes for smoking-induced diseases *NCAM1* and *IREB2*. Our findings revealed the role of cerebral WM in the maintenance of the complex addiction and provided potential genetic targets for future research in examining how changes in WM integrity contribute to the nicotine effects on the brain.

## Introduction

Tobacco smoking is a complex addictive behavior and is the chief modifiable causal factor for cancer, coronary and cerebrovascular disorders, chronic obstructive pulmonary disorder, hypertension, and many other illnesses (McBride, 1992; Ziedonis et al., 2008). Additive genetic factors explain up to ∼75% of the variance on traits that quantify nicotine dependence and smoking-related behaviors in population studies, thus supporting the hypothesis of a strong genetic susceptibility for nicotine addiction (Kendler et al., 1999; Vink et al., 2005). Genome-wide association studies (GWASs) localized and replicated multiple genetic variants conferring susceptibility to smoking/nicotine addiction including those regulating nicotinic acetylcholine receptors (nAChRs) (True et al., 1999; Erzurumluoglu et al., 2019). However, its neurogenetic mechanisms remain unknown. Neuroimaging studies reported observational (Zhang et al., 2010; Gons et al., 2011; Gray et al., 2020) and direct associations (Kochunov et al., 2013) between smoking and nicotine administration and white matter (WM) integrity, assessed by the fractional anisotropy (FA) of water diffusion in diffusion tensor imaging (DTI) (Kochunov et al., 2007). Cerebral WM is hypothesized to be involved in both positive and negative reinforcement mechanisms of nicotine addiction (Benowitz, 2010). The positive effect of nicotine is associated with cognitive and mood enhancement, which could be driven through the transient elevation in WM integrity following nicotine administration (Perkins, 1999; Kochunov et al., 2013). The nAChRs have been found in the cerebral WM of both human and nonhuman primates in the previous PET ligand and histological binding studies (Fujita et al., 2003; Ding et al., 2004; Pimlott et al., 2004; Hillmer et al., 2011). Their functions have been studied in peripheral (Lang et al., 2003) and central (Zhang et al., 2011a) nervous systems. The negative effect of nicotine in avoiding withdrawal symptoms may also be caused by reduction in WM integrity due to cerebrovascular and neurodegenerative risks associated with heavy chronic smoking (Hudkins et al., 2010; Kim et al., 2010; Gons et al., 2011; Liao et al., 2011; Zhang et al., 2011a).

**FIGURE 1:**
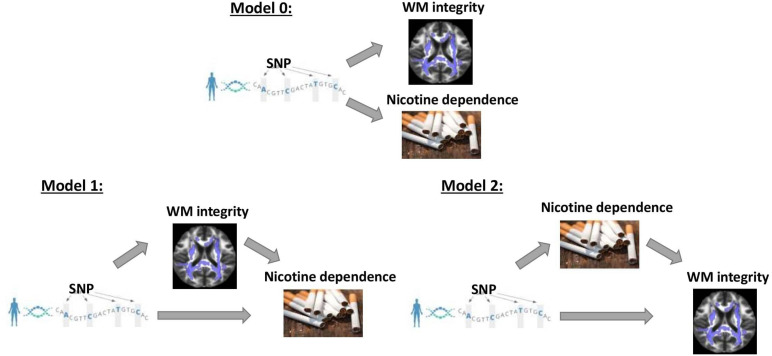
Three competing vertical and horizontal pleiotropy pathways proposed to understand the causal relationship between genetics, white matter integrity, and nicotine dependence. Model 0 represents a horizontal pleiotropic relationship, while models 1 and 2 represent vertical pleiotropic relationships.

Vertical and horizontal pleiotropic pathways are chief causal mechanisms explaining the phenomenon of genetic variants affecting multiple traits (Tyler et al., 2009; Paaby and Rockman, 2013; Jordan et al., 2019). Vertical pleiotropy is observed when a trait influenced by genetic factors has, in turn, influenced another trait by acting as a mediator. Horizontal pleiotropy refers to two traits being independently influenced by the same genetic factors. In the first vertical pleiotropic pathway (Figure 1: model 1), we assume that genetic factors underpinning susceptibility to nicotine dependence do so through alterations in WM integrity. In the second vertical pleiotropic pathway (Figure 1: model 2), we alternatively hypothesize that genetic factors underpin nicotine addiction directly, and the reduction in WM integrity occurs due to a multitude of harmful effects of chronic or heavy smoking. Lastly, we also test horizontal pleiotropy (Figure 1: model 0) where genetic factors affect both WM integrity and nicotine addiction independently. The proposed models are complementary to the widely used Mendelian randomization (MR) models that estimate the causal influence between the traits by relaxing the conditional independence assumption in instrumental variable (IV) analysis (Davey Smith and Hemani, 2014; Hemani et al., 2018a,b). We then develop rigorous analytical approaches to determine and validate the best causal model for pleiotropic genetic variants associated with both WM integrity and nicotine addiction.

We analyzed two smoking phenotypes for their biological relevance to nicotine dependence, smoking status (SS) and cigarettes per day (CPD), to test the proposed causal pathways in large-scale epidemiological data from the UK Biobank (UKBB). We used FA to measure WM integrity. Smoking (being current smoker or having higher CPD) was found to be associated with reduced WM integrity in multiple brain regions. Our data showed that the genetic effects on FA and the two smoking phenotypes were not independent, so the horizontal pleiotropy does not hold. Since model 1 and model 2 with a vertical pleiotropic relationship are two mutually exclusive causal pathways, we performed mediation analysis and used Bayes factor to select the optimal model. We identified 272 pleiotropic variants associated with both SS and FA whose effects on SS were mediated by FA (model 1 preferred). On the contrary, 22 pleiotropic variants were found to be associated with both CPD and FA, where CPD acts as a mediator for the genetic effects on FA (model 2 preferred). The identified variants mainly reside in two genes *NCAM1* and *IREB2*. Their relationship to the smoking-induced brain mechanisms will need to be further examined for their functionality in future studies.

## Materials and Methods

### UK Biobank Cohort

The data used to test our causal pathways are from the UKBB, a large prospective study that recruited ∼500,000 participants aged between 40 and 69 years in 2006–2010 in 22 assessment centers throughout the United Kingdom (Sudlow et al., 2015). UKBB data consist of phenotypic, genotypic, and imaging details about its participants collected from questionnaires, physical measures, multimodal imaging, genome-wide genotyping, and longitudinal follow-up for health-associated outcomes (Sudlow et al., 2015). In our analysis, UKBB data from all sites and all phases are included. UKBB carried out an automated processing pipeline for sample retrieval, data collection, and quality control to convert raw data to reliable processed data for use by all researchers (UK Biobank, 2007; Alfaro-Almagro et al., 2018; Bycroft et al., 2018). We restricted our analysis to only participants with white ethnicity backgrounds (British, Irish, and any other white background) and with both genotype and nicotine dependence phenotype data available. For causal pathway analysis, we further narrowed down to participants who have genotype, nicotine dependence, and WM integrity phenotype data available. The number of participants included at each analytic step is summarized in Supplementary Figure 1.

### Nicotine Dependence-Related Smoking Phenotypes

Supplementary Table 1 summarizes the number of participants by smoking-related phenotype codes in UKBB. We chose to analyze the following two phenotypes due to their biological relevance to nicotine dependence (Donny et al., 2008; Benowitz, 2010):

(1) Smoking Status (SS; current vs. never smokers). SS was a binary trait describing subjects who were either current smokers or never smokers (past smokers excluded). Current and never smokers were defined using phenotype code 20116 (smoking status) in UKBB.
(2) Cigarettes Per Day (CPD; average number of cigarettes smoked per day by participants who are either current or past smokers). CPD was a quantitative trait that measures the heaviness of smoking among ever smokers (never smokers excluded). It was defined using phenotype codes 2887 (number of cigarettes previously smoked daily), 3456 [number of cigarettes currently smoked daily), and 6183 (number of cigarettes previously smoked daily (current cigar/pipe smokers)] in UKBB. The CPD values of participants who smoked less than one CPD were recoded to 0; and CPD values of those who smoked more than 60 CPD were recoded to 60.

Note that SS and CPD are both related to nicotine dependence but target different smoker categories. SS focuses on the difference between current smokers and never smokers, while CPD focuses on the heaviness of smoking among current or past smokers.

### White Matter Integrity Phenotype Measured by Fractional Anisotropy

The UKBB consists of multimodal braining imaging data covering structural, functional, and diffusion imaging (Miller et al., 2016). In this study, we concentrated on the WM FA measure derived from diffusion MRI data, a common measure of WM integrity whose association with smoking addiction behavior has been reported in previous studies (Gogliettino et al., 2016). The UKBB database provides 40 FA measures from multiple brain regions (full region names listed in Supplementary Table 2), including the inferior cerebellar peduncle (ICP), genu of the corpus callosum (GCC), body of the corpus callosum (BCC), splenium of the corpus callosum (SCC), fornix (FX), corticospinal tract (CST) (mean/right/left), anterior limb of the internal capsule (ALIC) (right/left), posterior limb of the internal capsule (PLIC) (right/left), retrolenticular part of the internal capsule (RLIC) (right/left), anterior corona radiata (ACR) (right/left), superior corona radiata (SCR) (right/left), posterior corona radiata (PCR) (right/left), posterior thalamic radiation (PTR) (right/left), sagittal striatum (SS) (right/left), external capsule (EX) (right/left), cingulum cingulate gyrus (CGC) (right/left), cingulum hippocampus (CHG) (right/left), fornix cres+stria terminalis (FXST) (right/left), superior longitudinal fasciculus (SLF) (right/left), superior fronto-occipital fasciculus (SFO) (right/left), uncinate fasciculus (UN) (right/left), and tapetum (TAP) (right/left). The data were preprocessed using pipeline similar to that developed by Enhancing Neuro Imaging Genetics Meta Analysis (ENIGMA) consortium (Thompson et al., 2014). Before conducting the causal pathway analysis for each FA, we further performed univariate association analysis by regressing FA measures on the two smoking phenotypes and kept only those FA measures with lower values among current smokers or negatively associated with CPD ($\hat{\beta }<0$, *p* < 0.05), motivated by the findings from previous studies (Savjani et al., 2014; Umene-Nakano et al., 2014; Gray et al., 2020).

### Genotype Data and Genome-Wide Association Study (GWAS)

The genotype data of UKBB cohort came from two platforms, Affymetrix UK BiLEVE Axiom and UKBB Axiom^®^ arrays, which captured over 90 million single-nucleotide variants (SNVs) of ∼500,000 subjects (Bycroft et al., 2017). We first removed variants with minor allele frequency (MAF) below 0.01, Hardy–Weinberg equilibrium *p*-value below 0.001, and missing genotype rate at 5% and removed individuals with more than 2% missing genotypes. We conducted principal component analysis (PCA) method to adjust for population stratification and chose the top 10 principle components (PCs) as recommended by PLINK (version 2.0)^1^ (Chang et al., 2015) and in previous studies (Feng et al., 2009; Kang et al., 2009; Price et al., 2010; Warren et al., 2017). We then performed GWAS separately on the two smoking phenotypes SS and CPD adjusting for age, gender, body mass index, genotyping chip type, and the acquired top 10 PCs using PLINK (version 1.9^2^) (Chang et al., 2015). The most significantly associated loci (i.e., *p* < 5 × 10^–8^) were used for the causal pathway analysis. Considering the strong linkage disequilibrium (LD) of neighboring single-nucleotide polymorphisms (SNPs), we also included SNPs in the genomic regions ± 250 kb around the peak boundaries (Supplementary Figures 2–7). GWAS was not performed on FA measures, which is underpowered due to the relatively small number of participants having neuroimaging data available. In the causal pathway analysis, we further selected pleiotropic SNPs that are associated with both smoking phenotypes and FA measures.

### Causal Pathway Discovery

Denote by G the genotype, M the FA measures, Y the smoking phenotypes (SS or CPD), and Z the potential covariates. In this study, we included age and gender as covariates to be adjusted in the model. Given the directed graph structures in Figure 1, we can represent the three competing models by factorizing their joint distributions:

Model0:Pr(M,Y|G,Z)=Pr(M|(G,Z))Pr(Y|(G,Z))

Model1:Pr(M,Y|G,Z)=Pr(M|(G,Z))Pr(Y|(M,G,Z))

Model2:Pr(M,Y|G,Z)=Pr(Y|(G,Z))Pr(M|(Y,G,Z))

Model 0 assumes genetics to be a common cause of both FA and smoking phenotypes independently (“SNP → Smoking,” “SNP → FA” and FA ⊥ Smoking given SNP and confounders) and represents a horizontal pleiotropic relationship. Model 1 and model 2 are two alternative mediation models that represent a vertical pleiotropic relationship. In model 1 (“SNP → FA → Smoking”), FA measures are regarded as the mediators that mediate the effect of SNPs on smoking. In contrast, model 2 (“SNP → Smoking → FA”) considers the long-term effect of chronic smoking on the brain structure and regards smoking as the mediator that mediates the effect of SNPs on FA.

We performed automatic causal pathway discovery analysis (Heckerman et al., 1999; Spirtes and Zhang, 2016; Glymour et al., 2019) to identify the optimal pleiotropic pathway. Our analyses started by identifying the potentially pleiotropic variants of FA and smoking for each FA measure separately. We then evaluated the association between each FA measure and smoking given the SNP effects to distinguish horizontal pleiotropy from vertical pleiotropy. For variants with a vertical pleiotropic relationship, we further conducted causal mediation analysis to choose the best mediation model that explains the relationship between SNP, FA, and smoking. Below, we describe the analytical steps in details.

#### Step 1: Identification of Pleiotropic Variants

Suppose we start with a set of *g*_*0*_ SNPs gained by GWAS for *n* subjects. Let *G*_*ij*_ denote the genotype of the *i*th subject in the *j*th SNP (1≤*i* ≤*n*, *j* ∈ *g*_0_), *M*_*il*_ denote the *l*th continuous FA measure (1≤ *l* ≤40) of *i*th subject, *Y*_*i*_ denote the SS/CPD value, and *Z*_*i*_ denote the covariates of the *i*th subject. We assume an additive genetic model, and let *G*_*ij*_ = 0, 1,or, 2 represent the number of copies of minor alleles. In the first step, we look for SNPs that are associated with both FA measures and SS/CPD (i.e., potentially pleiotropic variants). Notably, this step is also a necessary condition to establish mediation for both model 1 and model 2, where the mediator and the outcome are simply switched in the two models (Judd and Kenny, 1981; James and Brett, 1984; Baron and Kenny, 1986). First, we fit a linear regression model on each SNP for each FA measure separately adjusting for the covariates Z to look for SNP–FA association. Then we fit a logistic regression or a linear regression model on each SNP for SS or CPD, adjusting for the covariates Z to look for SNP–smoking association:

(1a) Regress M on G and Z:

$$M_{i\,l}=\alpha_{1}+\beta_{1\,j}\,G_{i\,j}+\gamma_{1}\,Z_{i}+\varepsilon_{1\,i},\varepsilon_{1\,i}∼N\,\left(0,\sigma_{1}^{2}\right),$$

$$\,1\leq i\leq n,1\leq l\leq 40,j\in g_{0}$$

(1b) Regress Y on G and Z:

$$Y\mathrm{is}\mathrm{binary}\left(\mathrm{SS}\right):\text{logit}\left(P\left(Y_{i}=1\right)\right)=\alpha_{2}+\beta_{2\,j}G_{i\,j}+\gamma_{2}{\text{Z}}_{i},$$

$$\,1\leq i\leq n,j\in g_{0}$$

$$Y\,\mathrm{is}\,\mathrm{continuous}\,\left(\mathrm{CPD}\right):Y_{i}=\alpha_{2}+\beta_{2\,j}\,G_{i\,j}+\gamma_{2}\,Z_{i}+\varepsilon_{2\,i},\varepsilon_{2\,i}$$

$$∼N\,\left(0,\sigma_{2}^{2}\right),1\leq i\leq n,j\in g_{0}$$

where α_*1*_ and α_*2*_ are the intercepts, γ_*1*_ and γ_*2*_ are the effects of covariates, and β_*1j*_ and β_*2j*_ correspond to the genetic effect of the *j*th SNP on M and Y (subscript *l* was omitted for α_*1*_ and β_*1j*_ for simplicity). The cutoff for statistical significance is chosen to control for the overall false discovery rate (FDR < 0.15) in identifying the potentially pleiotropic variants in the shared subset that meets both SNP–FA and SNP–smoking association criteria (see Supplementary Methods). We then used the Functional Annotation of Variants—Online Resource (FAVOR) (Li et al., 2020) to annotate the identified pleiotropic variants for more biological insights.

#### Step 2: Distinguish Horizontal From Vertical Pleiotropy

Model 0 assumes a horizontal pleiotropic relationship, while models 1 and 2 assume a vertical pleiotropic relationship. The main feature of horizontal pleiotropy is that the two traits (smoking and FA) are independent given the SNP effect. In this step, we conducted association analysis between FA and SS/CPD conditioning on the SNP. If the conditional independence holds (conditional independence test *p* > 0.05), the variants demonstrate horizontal pleiotropy; otherwise, they demonstrate vertical pleiotropy.

#### Step 3: Selection of the Best Mediation Model for Vertical Pleiotropy

Model 1 and model 2 are two mutually exclusive mediation models under the vertical pleiotropy assumption. Mediation analysis investigates how a third variable affects the relation between two other variables and is a useful tool in discovering the hidden mechanism in many biological fields (MacKinnon et al., 2007). We conducted exploratory mediation analysis and selected the best mediation model for each pleiotropic SNP using Bayes factor criteria (Kass and Raftery, 1995; Denison et al., 2002; Bernardo and Smith, 2009). We validated the selected model by checking the major causality assumptions and testing and categorizing the mediation effects.

Suppose that the set of SNPs that meet the criteria of vertical pleiotropy from step 1 and 2 is *g*_1_, to determine the best mediation model from the two candidates, and we regress the outcome on both exposure variable and mediator for model 1 (outcome = SS/CPD) and model 2 (outcome = FA), respectively, adjusting for covariates Z:

(2a) Regress Y on G, M, and Z:

$$Y\mathrm{is}\mathrm{binary}\left(\mathrm{SS}\right):\text{logit}\left(P\left(Y_{i}=1\right)\right)=\alpha_{3}+\beta_{31\,j}G_{i\,j}+\beta_{32}M_{i\,l}$$

$$+\gamma_{3}\,Z_{i},\,1\leq i\leq n,\,1\leq l\leq 40,\,j\in g_{1}$$

$$Y\,\mathrm{is}\,\mathrm{continuous}\,\left(\mathrm{CPD}\right):Y_{i}=\alpha_{3}+\beta_{31\,j}\,G_{i\,j}+\beta_{32}\,M_{\text{i}\,l}+\gamma_{3}\,Z_{i}$$

$$+\varepsilon_{3\,i},\varepsilon_{3\,i}∼N\,\left(0,\sigma_{3}^{2}\right),1\leq i\leq n,1\leq l\leq 40,j\in g_{1}$$

(2b) Regress M on G, Y, and Z:

$$M_{i\,l}=\alpha_{4}+\beta_{41\,j}\,G_{i\,j}+\beta_{42}\,Y_{i}+\gamma_{4}\,Z_{i}+\varepsilon_{4\,i},\varepsilon_{4\,i}∼N\,\left(0,\sigma_{4}^{2}\right),$$

$$\,1\leq i\leq n,1\leq l\leq 40,j\in g_{1}$$

where α_*3*_ and α_*4*_ are the intercepts; γ_*3*_ and γ_*4*_ are the effects of covariates; β_*31j*_ and β_*41j*_ represent the direct effects of SNPs on outcomes Y and M in models 1 and 2, respectively; and β_1*j*_β_32_ and β_2*j*_β_42_ represent the indirect effects of SNPs on outcome via the mediators M or Y in models 1 and 2, respectively.

To select the mediation model that best explains the causal relationship for each SNP *j*, we propose to use the penalized likelihood Bayesian information criterion (BIC) score as a model selection criterion (Schwarz, 1978). By definition, $\text{BIC}=-2\,\text{log}\,\left(\hat{L}\right)+p\,\text{log}\,\left(n\right)$, where $\hat{L}$ denotes the maximized value of the likelihood, *p* is the number of parameters, and *n* is the sample size. Since the two mediation models have exactly the same number of parameters, we are directly comparing the maximum likelihoods of the two models. The maximum likelihoods of model 1 and model 2 can be derived from their joint distribution combining step 1 and step 2 (model 1: 1a+2a; model 2: 1b+2b), with the parameters evaluated at maximum likelihood estimation (MLE):

$$\mathrm{Model1}:\hat{L}\,\left(M\,1\right)=L\,\left(\hat{\alpha_{1}},\hat{\beta_{1\,j}},\hat{\gamma_{1}},\hat{\sigma_{1}^{2}}|M_{l},G_{j},Z\right)$$

$$\;\;L\,\left(\hat{\alpha_{3}},\hat{\beta_{31\,j}},\hat{\beta_{32\,j}},\hat{\gamma_{3}},\hat{\sigma_{3}^{2}}|Y,G_{j},M_{l},Z\right)$$

$$\mathrm{Model2}:\hat{L}\,\left(M\,2\right)=L\,\left(\hat{\alpha_{2}},\hat{\beta_{2\,j}},\hat{\gamma_{2}},\hat{\sigma_{2}^{2}}|Y,G_{j},Z\right)$$

$$\;\;L\,\left(\hat{\alpha_{4}},\hat{\beta_{41\,j}},\hat{\beta_{42\,j}}\,\hat{\gamma_{4}},\hat{\sigma_{4}^{2}}|M_{l},G_{j},Y,Z\right)$$

The BIC-based model selection performs exploratory mediation analysis to determine the favored mediation model for each potentially causal variant. We then validated the mediation model selected by carefully checking the causal mediation assumptions (see Supplementary Methods). Once the model assumptions are checked, the causal mediation has been established. For model 1, β_*31j*_ represents the direct effect, β_1*j*_β_32_ represents the indirect/mediation effect, and β_*2j*_ represents the total effect. For model 2, β_*41j*_ represents the direct effect, β_2*j*_β_42_ represents the indirect/mediation effect, and β_*1j*_ represents the total effect. Zhao et al. (2010) classified mediation into three types according to significance and direction of the direct effect when mediation effect is significant: when the direct effect is also significant and has the same sign, the mediation is called a complementary mediation; if they point to the opposite directions, the mediation is called a competitive mediation; lastly, if the direct effect is not significant, the mediation is indirect-only mediation. We followed this classification to interpret the final mediation results for each variant.

All statistical analyses were conducted using R (R Core Team, 2020). An R package “*mediation*” (Tingley et al., 2014) was used for model checking in mediation analysis.

## Results

### Genome-Wide Association Study and Selection of Smoking Associated Loci

**FIGURE 2:**
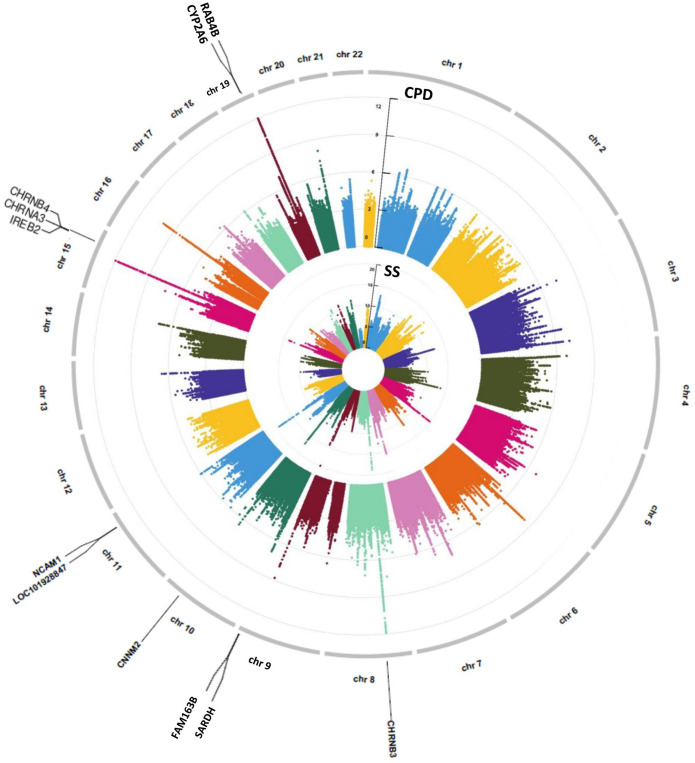
A concentric circular Manhattan plot of the GWAS results for smoking status (SS) and cigarette per day (CPD) for chromosomes 1–22. Each dot represents an SNV; *x*- and *y*-axis refer to genomic locations and –log10(*p*-value). The SNVs with –log10(*p*-value) are larger than 12 for chr15 and chr19 of CPD, and larger than 20 for chr9 and chr11 of SS were not included in the plot. The three highest signals mapped on genes *CHRNB3*, *CHRNA3*, *CHRNB4*, *IREB2*, *CYP2A6*, and *RAB4B* of CPD and the four highest signals mapped on genes *FAM163B*, *SARDH*, *CNNM2*, and *NCAM1* and lncRNA LOC101928847 of SS are labeled in the plot. GWAS, genome-wide association study; CPD, cigarette per day; SNV, single-nucleotide variant.

Genome-wide association study were conducted separately for SS and CPD (*N* = 293,759 and 142,202, respectively). Numerous important smoking behavior associated loci previously reported were reproduced in our study (Gelernter et al., 2006; Thorgeirsson et al., 2008; Keskitalo et al., 2009; Bloom et al., 2014; Bidwell et al., 2015a; Erzurumluoglu et al., 2019; Xu et al., 2020) as highlighted in the circular Manhattan plots (Figure 2). Notably, the significant loci identified for each of the two traits have little overlap, implying the different genetic bases of the two nicotine dependence-related smoking phenotypes. The loci associated with SS are mainly located in regions on chromosomes 9, 10, and 11 marked by genes *FAM163B*, *SARDH*, *CNNM2*, and *NCAM1* and non-coding RNA LOC101928847, while loci associated with CPD are located in regions on chromosomes 8, 15, and 19 marked by genes *CHRNB3*, *CHRNA3*, *IREB2*, and *RAB4B* (Table 1). The results validated the gene findings of smoking behaviors in previous GWASs (Ware et al., 2011; Lassi et al., 2016).

**TABLE 1:** The selected genomic regions for causal pathway analysis and representative marker genes.

CHR	Regions selected		Numbers of SNPs selected	Numbers of SNPs with GWAS *p*-value < 1e-8	Representative marker genes	References
	Start bp	End bp				
**Cigarette per day (CPD)**						
8	42,302,562	42,842,209	1,321	130	*CHRNB3*	Thorgeirsson et al. (2008); Erzurumluoglu et al. (2019)
15	78,635,394	79,163,637	1,523	1,016	*CHRNA3*, *CHRNB4*, *IREB2*	Keskitalo et al. (2009); Erzurumluoglu et al. (2019)
19	41,033,670	41,552,849	1,576	428	*RAB4B*, *CYP2A6*	Thorgeirsson et al. (2008); Bloom et al. (2014), Erzurumluoglu et al. (2019)
**Smoking status (SS)**						
9	136,192,141	136,724,472	1,889	47	*FAM163B*, *SARDH*	Furberg et al. (2010); Xu et al. (2020)
10	104,428,075	105,088,344	1,547	536	*CNNM2*	Erzurumluoglu et al. (2019); Xu et al. (2020)
11	112,580,002	113,399,158	2,392	252	LOC101928847, *NCAM1*	Gelernter et al. (2006); Bidwell et al. (2015a), Xu et al. (2020)

*SNPs, single-nucleotide polymorphisms; GWAS, genome-wide association study.*

Next, we performed causal pathway analysis on the significantly associated loci (*p* < 5e-8) for SS and CPD. Considering the strong LD among nearby loci, we also included loci in the extended genomic regions by 250 kb both upstream and downstream of the peak regions (Supplementary Figures 2–7). This covers genomic regions including a total of 5,828 SNPs for SS and 4,420 SNPs for CPD (Table 1). In the causal pathway analysis stage, we focused on participants who have genotype, FA measure, and smoking phenotype data available (*N* = 23,624 for SS and *N* = 8,830 for CPD) and further narrowed down the analysis to pleiotropic variants that were also associated with FA measures (see Methods of Causal pathway analysis Step 1).

### Causal Pathway Analysis for Smoking Status

**FIGURE 3:**
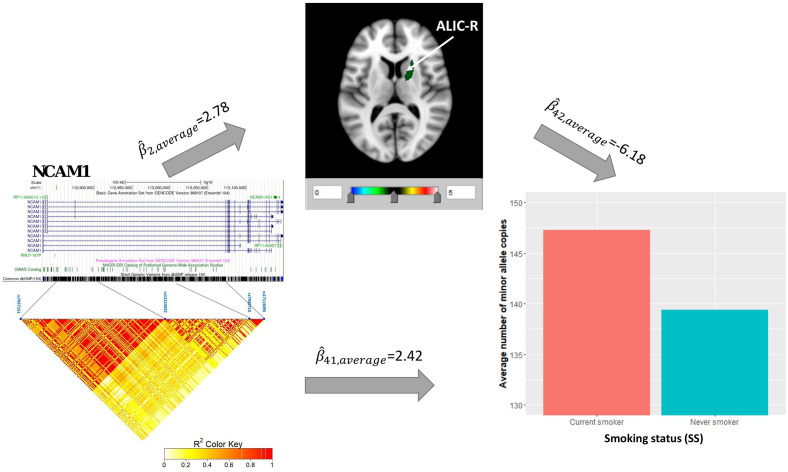
ALIC-R mediates the genetic effects of 244 variants located within *NCAM1* (chr11: 112,835,024–113,060,660) on SS. Exact locations of the 244 variants on the genome and their pairwise LD scores are shown. Bar chart shows the average number of minor allele copies for 244 variants among two groups of subjects to describe the current smokers have more copies of minor alleles than never smokers. Colors in the brain images denote the average –log10(*p*-value) of FA association with the 244 variants. The direct (${\hat{\beta }}_{41}$) and indirect effects (${\hat{\beta }}_{2}\,{\hat{\beta }}_{42}$) are averaged over all SNPs. ALIC-R, right tract of the anterior limb of the internal capsule; LD, linkage disequilibrium; FA, fractional anisotropy; SNPs, single-nucleotide polymorphisms.

Univariate association analysis found 29 FA measures from various brain regions that show significantly lower FA values among current smokers than never smokers ($\hat{\beta }<0$, *p* < 0.05; Supplementary Table 3), supporting the findings in previous literature (Savjani et al., 2014; Umene-Nakano et al., 2014; Gray et al., 2020). We proceeded with the causal pathway analysis for each of these 29 FA measures. Only the FA measure in the right tract of the ALIC (ALIC-R) had pleiotropic variants that passed the statistical significance thresholds (overall FDR < 0.15); thus, we continued with this FA measure only in the subsequent steps. We observed 272 SNPs having a pleiotropic effect on SS and ALIC-R (detailed annotation information from FAVOR is included in Supplementary Table 5). The significant association between ALIC-R and SS held given the genetic effects of any of the SNPs, implying a vertical pleiotropic relationship (Supplementary Table 4), so we no longer proceed with model 0. In comparing the two mediation models of vertical pleiotropy, model 1 where FA mediates the genetic effect on SS is favored. Key mediation assumptions were checked for the chosen model. A majority of those variants (244 out of 272) resided in gene *NCAM1* (Supplementary Table 5). The role of *NCAM1* in addiction has been found in recent studies (Bidwell et al., 2015b; Muskiewicz et al., 2018). Zhu et al. (2016) discussed about two possible explanations for an observed association between genotype and trait, including true causality or strong LD with true causal variants in the same locus. Here, we did not distinguish the 244 variants for true causality vs. linkage and treated all of them as candidate causal variants. Figure 3 shows how these SNPs impacted SS via ALIC-R. Current smokers carry a significantly larger number of minor allele copies of these SNPs than never smokers on average (${\hat{\beta }}_{41}$ > 0). The indirect effect of carrying more minor alleles via FA in ALIC-R is in the opposite direction of the direct effect (${\hat{\beta }}_{2}\,{\hat{\beta }}_{42}$ < 0), thus regarded as competitive mediation (see Supplementary Table 6 for a complete mediation analysis results).

### Causal Pathway Discovery for Cigarette per Day

**FIGURE 4:**
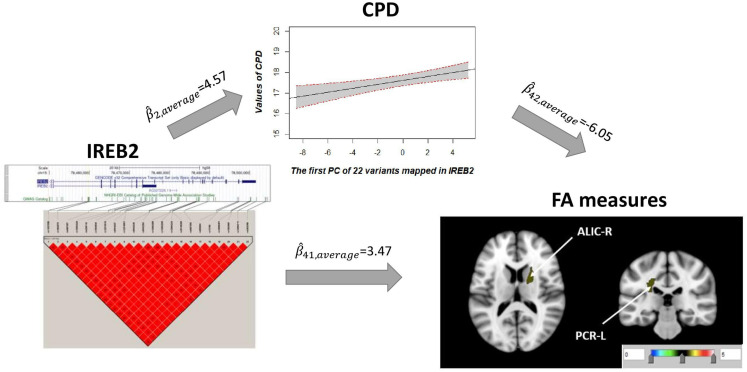
CPD mediates the genetic effects of 22 variants located within *IREB2* (chr15: 78,459,619–78,503,762) on two regional FA measures (ALIC-R and PCR-L). Exact locations of the 22 variants on the genome and their pairwise LD scores are shown. Due to the strong LD relationship among the variants, CPD was regressed on the first PC of 22 variants to describe the association of CPD vs. genetics. Colors in the brain images denote the average –log10(*p*-value) of FA association with the 22 variants. The direct (${\hat{\beta }}_{41}$) and indirect effects (${\hat{\beta }}_{2}\,{\hat{\beta }}_{42}$) are averaged over all SNPs and FA measures. CPD, cigarette per day; FA, fractional anisotropy; ALIC-R, right tract of the anterior limb of the internal capsule; PCR-L, left posterior corona radiata; LD, linkage disequilibrium; SNPs, single-nucleotide polymorphisms.

Univariate association found 29 FA measures that show significantly negative association with CPD ($\hat{\beta }$ < 0, *p* < 0.05; Supplementary Table 3). Among these 29 FA measures, only two FA measures in the ALIC-R and left tract of the PCR (PCR-L) had pleiotropic variants that passed the statistical significance thresholds (overall FDR < 0.15); thus, we continued with these two FA measures only in the subsequent steps. We observed 22 SNPs having a pleiotropic effect on CPD and FA measures ALIC-R and PCR-L (Supplementary Table 5). CPD and FA measures are dependent on each other given the genetic effects (Supplementary Table 4), implying a vertical pleiotropic relationship. Model 2 (CPD mediates the genetic effect on FA) was chosen as the best mediation model for these 22 SNPs, and the key mediation assumptions were checked for the chosen model. These variants are located in the exonic, intronic, and untranslated regions of gene *IREB2* (Supplementary Table 5). Figure 4 shows how these SNPs impacted the two regional FA measures ALIC-R and PCR-L via CPD. The minor alleles of these SNPs appear to have a protective direct effect on brain structure (${\hat{\beta }}_{41}$ > 0), while exerting an adverse effect associating with higher CPD (${\hat{\beta }}_{2}\,{\hat{\beta }}_{42}$ < 0), regarded as competitive mediation (see Supplementary Table 6 for a complete mediation analysis results).

## Discussion

In this study, we used novel causative imaging genetics analyses to test neurogenetic mechanisms of nicotine dependence through altered WM integrity. We hypothesized and tested three pleiotropic models to explain the complex causal relationship among genetics, WM integrity, and nicotine dependence. Our GWAS on SS and CPD identified two different sets of associated genetic variants including many reported to be related to smoking in previous large-scale GWASs or meta-analyses (Hardin et al., 2012; Wain et al., 2015; Zeng et al., 2020). We also found smoking (being current smoker or having higher CPD) to be associated with lower WM integrity measured by FA in multiple brain regions. The causal pathway analysis identified 272 pleiotropic SNPs associated with FA in ALIC(R) and SS, and 22 pleiotropic SNPs associated with FA in ALIC(R) and PCR(L) and CPD. These SNPs are mainly located in genes *NCAM1* and *IREB2*. *NCAM1* was found to influence risk of nicotine addiction (Gelernter et al., 2006; Muskiewicz et al., 2018). *IREB2*, which regulates iron mechanism in the cell, was a susceptibility gene for both neurodegeneration and smoking-induced diseases (DeMeo et al., 2009; Cooper et al., 2019). Interestingly, these two sets of SNPs favored different vertical pleiotropic pathways: Gene → FA → SS vs. Gene → CPD → FA. The basic genetic components of addiction might have produced a pattern change in WM among smokers, reinforcing the addiction behavior. Chronic severe smoking (reflected in, e.g., CPD) will have negative impact to overall health, which in turn reduces the WM integrity.

We have used an imaging genetics approach to test the neurogenetic mechanisms. Traditional imaging genetics treat neuroimaging as the mediator of the genetic effect on behavior through a unidirectional model (Meyer-Lindenberg and Weinberger, 2006; Bogdan et al., 2017). We use novel causal mediation models to evaluate vertical and horizontal pleiotropy pathways, SNP → behavior → brain vs. SNP → brain → behavior vs. SNP → behavior and SNP → brain, in understanding the neurogenetic mechanism of nicotine addiction behavior. Constraint-based methods and score-based methods are two main categories of conventional causal discovery methods (Glymour et al., 2019). Constraint-based methods identified causal links by conducting conditional independence tests, while score-based methods selected the model with the optimal score from multiple candidate causal models (Spirtes and Zhang, 2016). Here, we proposed to perform conditional independence test to distinguish vertical pleiotropy from horizontal pleiotropy, and we used a BIC score-based method to select the optimal model with the larger score from the two competing vertical pleiotropic models represented by directed acyclic graph (DAG). Such hybrid approach exploited principled ways to combine advantages of both methods and was a computationally efficient strategy to learn the causal structure in a wide range of real-life applications (Wong et al., 2002; Tsamardinos et al., 2006; Scutari et al., 2018). In our data, such an approach indeed resulted in a DAG with higher likelihood values.

Vertical pleiotropy and horizontal pleiotropy are two competing types of pleiotropy in complex polygenic traits (Paaby and Rockman, 2013). Conventional MR framework treats pleiotropic genetic factors as IV to elucidate how one phenotype (modifiable exposure) causally relates to another phenotype (the outcome) (Davey Smith and Hemani, 2014), but it only considers the vertical pleiotropy with no direct causal link between SNP and outcome (known as exclusion restriction assumption) (Hemani et al., 2018a). We developed a model that evaluates both vertical and horizontal pleiotropy pathways simultaneously and used a rigorous likelihood-based approach to determine the optimal model. We found a significant direct effect of SNPs on the outcomes using our data, which violates the exclusion restriction assumption (Davey Smith and Hemani, 2014). Such extension from the MR framework to tolerate SNPs violating the exclusion restriction assumption of IV analysis has also been seen in recent genetic literature (Bowden et al., 2015; Verbanck et al., 2018). In addition, to establish causality, we also carefully checked the main causal mediation model assumptions of the optimal model.

The biophysical mechanisms that link smoking and lower WM integrity are unknown. Most studies in chronic and heavy smokers reported reduced FA values when compared with those in nonsmokers (Swan and Lessov-Schlaggar, 2007; Hudkins et al., 2010; Kim et al., 2010; Cullen et al., 2011; Gons et al., 2011; Liao et al., 2011; Zhang et al., 2011a, b). Absolute WM FA values are sensitive to many parameters including myelin content, intra-voxel axonal crossing, and axonal fiber density and diameter (Beaulieu, 2002). However, long-term changes in regional FA values were shown to be mainly (*r* > 0.8) driven by changes in regional cerebral myelin concentrations and myelin packing (Song et al., 2003, 2005; Madler et al., 2008). Our findings strongly implicate cerebral WM in the maintenance of this complex addiction and provide genetic targets for further analyses. This finding should encourage future research to examine how changes in WM integrity may or may not contribute to the overall nicotine effects on brain and cognition. Heavy chronic smoking increases the risk of the development of abnormalities in vascular endothelial morphology and function, which may cause cerebral perfusion leading to poorer neurocognition (Pittilo, 2000). Additionally, chronic smoking can impact the vasomotor reactivity/responsivity of the cerebrovascular through upregulation of Ca^2+^ channels and/or modulation of nitric oxide, resulting in the reduction of regional cerebral blood flow (Zubieta et al., 2001; Domino et al., 2004) and the development of WM disease (Liao et al., 1997; Ding et al., 2003; Jeerakathil et al., 2004).

Our study population consists of individuals with white ethnic background, and we pooled the UKBB data from different assessment sites and phases together in the analyses without considering the heterogeneity across sites and phases. Future studies are needed to evaluate the generalization of our conclusion to individuals from other races or ethnicities and to assess whether the conclusions are related to site and phase effects. Our study focused on the most significant variants from GWAS for the causal pathway analysis. Considering the high dimensionality of SNPs and the complex polygenic architecture of both smoking and WM integrity, SNPs with weak signals may not be identified. Further investigation in large independent cohorts is needed to validate current causal analysis results and provide a full picture of the complex genetic architecture of smoking and brain structure, which will in turn improve our understanding of the neurogenetic mechanism of nicotine addiction behavior.

## Data Availability Statement

The raw genetic and phenotypic data used in the current study are available from the UK Biobank (UKBB), which can be accessed via https://www.ukbiobank.ac.uk/.

## Author Contributions

ZY, CM, and SL performed the analysis and wrote the manuscript. TM, SC, and PK supervised the project and took the lead in editing the manuscript. KH, SG, YM, LH, PT, NJ, AA, HG, LS, and TN contributed to the manuscript writing and polishing. All authors provided critical feedback and helped to shape the research, analysis, and manuscript.

## Conflict of Interest

The authors declare that the research was conducted in the absence of any commercial or financial relationships that could be construed as a potential conflict of interest.

## Publisher’s Note

All claims expressed in this article are solely those of the authors and do not necessarily represent those of their affiliated organizations, or those of the publisher, the editors and the reviewers. Any product that may be evaluated in this article, or claim that may be made by its manufacturer, is not guaranteed or endorsed by the publisher.

## Footnotes

1
www.cog-genomics.org/plink/2.0/

2
www.cog-genomics.org/plink/1.9/
