# Supplementary material for: White Matter Integrity and Nicotine Dependence: Evaluating Vertical and Horizontal Pleiotropy
Source: Front Neurosci. 2021 Oct 14;15:738037. doi: 10.3389/fnins.2021.738037 (PMC8551454; doi:10.3389/fnins.2021.738037)
Supplement: Supplementary file 1 [file Data_Sheet_1.docx]

Supplementary Material

**1. Supplementary Methods**

**1.1 Multiple comparison procedure in the selection of pleiotropic loci**

In step 1, tests of genetic effects in regression functions (1a) and (1b) are considered to claim SNP findings. Bogomolov and Heller (2013) first proposed a theoretically justified multiple testing procedure to control for the overall false discovery rate (FDR) in the identification of findings that replicate from a high-dimensional study to another. Sampson et al. (2018) further proposed a multiple comparison procedure to control for overall FDR when testing multiple mediators in mediation analysis. In this study, we follow a similar idea and propose a multiple comparison procedure to control for the overall FDR when testing multiple exposures in mediation analysis. Suppose the targeted overall FDR we wish to control is α, we first set $t_{1}$= α/2 as a threshold for a significant linear relationship between SNP and CPD, and define $\omega_{S_{1}}=\{j:P_{1j}\leq t_{1}\}$ and $S_{1}=C(\omega_{S_{1}})$ where $P_{1j}$ is the p-value for the association test between SNP j and CPD and C(.) is the number of elements in $\omega$. Similarly, set $t_{2}$=α/2 as a threshold for a significant linear association between SNP and FA measure, and define $\omega_{S_{2}}=\{j:P_{2j}\leq t_{2}\}$ and $S_{2}=C(\omega_{S_{2}})$. We further compute a subset-adjusted P-value $P_{Sj}=2max(S_{2}P_{1j},S_{1}P_{2j})$ if $P_{1j}\leq t_{1}$ and $P_{2j}\leq t_{2}$, and 1 otherwise. We claim SNP j as a pleiotropic SNP in subsequent analysis if $P_{Dj}={min}_{j':P_{Sj'}\geq P_{sj}}\frac{P_{sj^{'}}}{rank\left( P_{sj^{'}} \right)}$ ≤ α, where $P_{Dj}$ is the FDR-adjusted P-value and rank($P_{Sj}$) is the rank of $P_{Sj}$ for SNP j ∈ $\omega_{S_{1}}$⋂ $\omega_{S_{2}}$.

**1.2 Causal mediation assumptions**

As a standard in the causal inference literature (Hernán and Robins 2010, Imai et al. 2010, Imbens and Rubin 2015), we made the following assumptions for the identifiability of the causal mediation effect:

(A1) Stable Unit Treatment Value Assumption (SUTVA): M=m implies Y=Y(m) for model 1 and Y=y implies M=M(y) for model 2.

(A2) (Weak) Ignorability: $Y\left( m \right)\perp M|G$ for model 1, $M\left( y \right)\perp Y|G$ for model 2.

(A3) Positivity: the probability of being assigned to each level of M and Y within their defined range is greater than zero.

(A4) Sequential ignorability: $\{Y_{i}\left( g^{'},m \right),M_{i}(g)\}\perp G_{i}|Z_{i}=z$; $Y_{i}\left( g^{'},m \right)\perp M_{i}(g)|G_{i}=g,Z_{i}=z$ for model 1, and $\{M_{i}\left( g^{'},y \right),Y_{i}(g)\}\perp G_{i}|Z_{i}=z$; $M_{i}\left( g^{'},y \right)\perp Y_{i}(g)|G_{i}=g,Z_{i}=z$ for model 2.

(A1)-(A3) are necessary to establish the direct causal link between M and Y and are generally untestable. (A4) is specific to the causal mediation effect. It is a strong assumption, we used the R package “mediation” (Tingley et al. 2014) to perform sensitivity analysis to check the assumption.

For confounding, we further made the following four main assumptions following the literature in mediation analysis (Tyler 2016):

(C1) Confounding is controlled in the exposure-outcome association

(C2) Confounding is controlled in the mediator-outcome association

(C3) Confounding is controlled in the exposure-mediator association

(C4) No mediator-outcome confounder is by itself affected by the exposure

Assumptions (C1) and (C3) are checked in step 1 where we include the potential confounders (in our example, Z include age and gender). Assumption (C2) is checked in step 2. We also further check (C4) here and consider whether the confounders in step 2 (e.g., age and gender) are themselves related to the causal variants of CPD/SS.

# Supplementary Figures and Tables

## Supplementary Figures


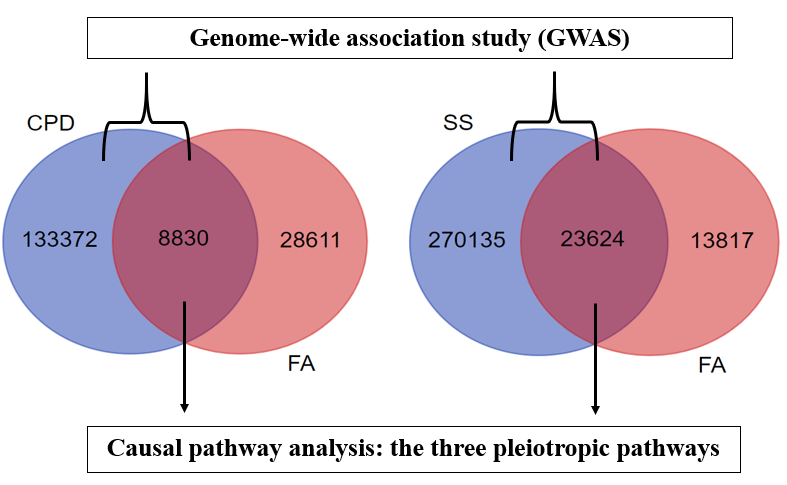


**Figure S1.** Overview of participants selection in our study.


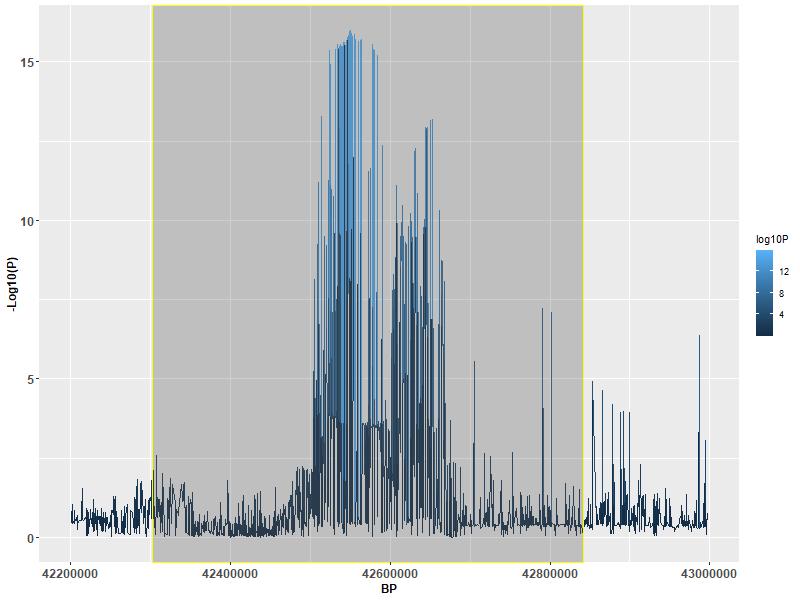


**Figure S2.** Manhattan plot of the region highlighted in grey strongly associated with CPD on chromosome 8 including 1321 SNPs located from chr8:42302562 to chr8:42842209 selected for causal pathway analysis.


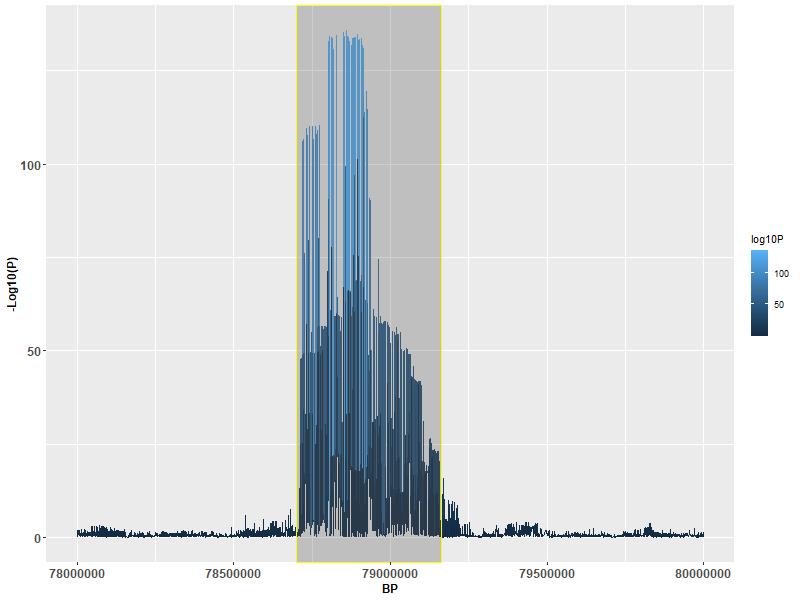


**Figure S3.** Manhattan plot of the region highlighted in grey strongly associated with CPD on chromosome 15 including 1523 SNPs located from chr15:78635394 to chr15:79163637 selected for causal pathway analysis.


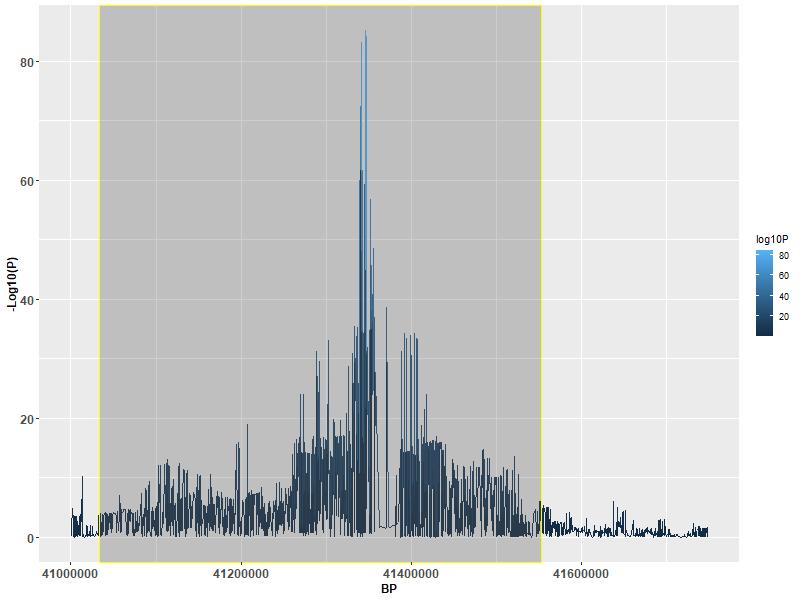


**Figure S4.** Manhattan plot of the region highlighted in grey strongly associated with CPD on chromosome 19 including 1576 SNPs located from chr19:41033670 to chr19:41552849 selected for causal pathway analysis.


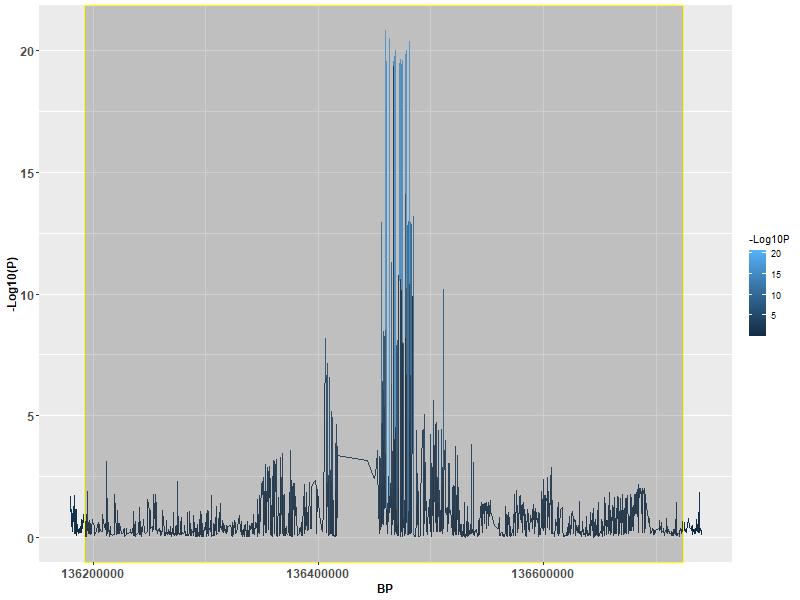


**Figure S5.** Manhattan plot of the region highlighted in grey strongly associated with smoking status on chromosome 9 including 1889 SNPs located from chr9:136192141 to chr9:136724472 selected for causal pathway analysis.


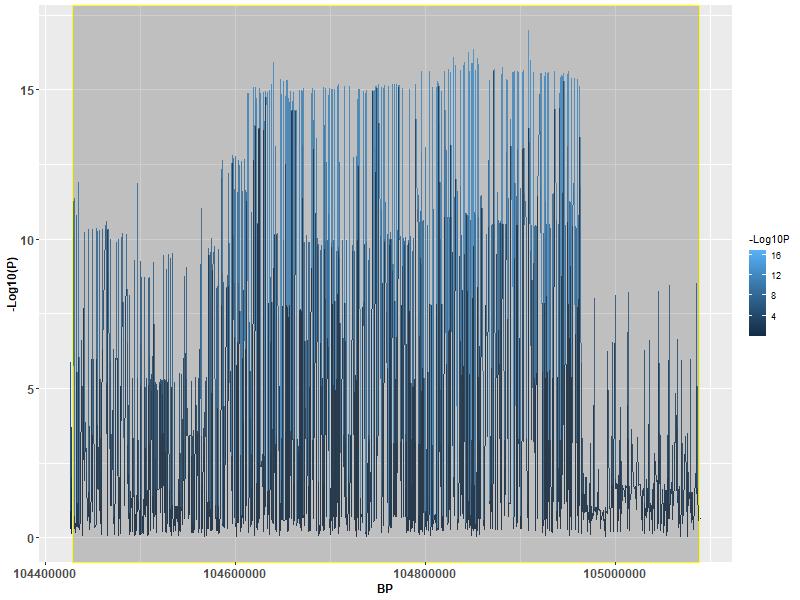


**Figure S6.** Manhattan plot of the region highlighted in grey strongly associated with smoking status on chromosome 10 including 1547 SNPs located from chr10:104428075 to chr10:105088344 selected for causal pathway analysis.


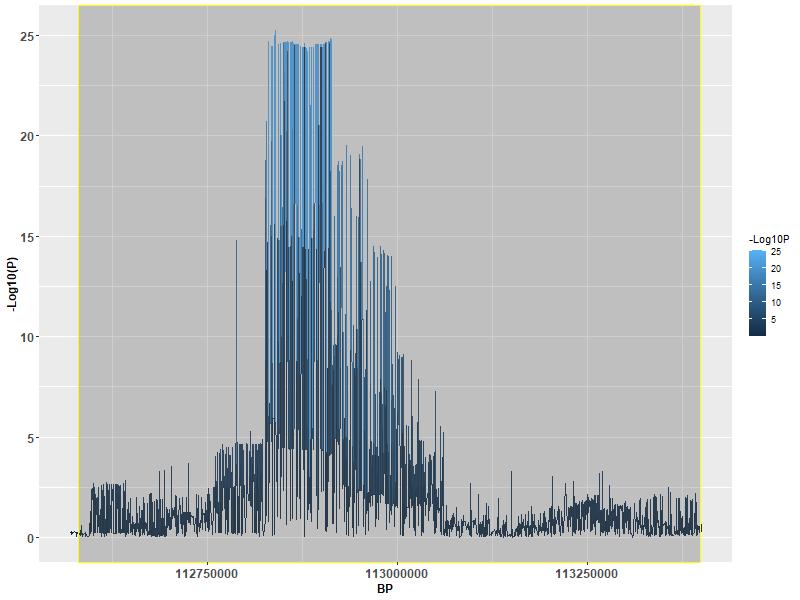


**Figure S7.** Manhattan plot of the region highlighted in grey strongly associated with smoking status on chromosome 11 including 2392 SNPs located from chr11:112580002 to chr11:113399158 selected for causal pathway analysis.

# Supplementary Tables

**Table S1.** Summary of number of participants by smoking-related phenotypes and covariates in UKBB used for GWAS and causal pathway discovery analysis, respectively.

| **GWAS analysis** | | | |
| --- | --- | --- | --- |
| Smoking status (SS) | Count | Cigarette per day (CPD) | Count |
| Never | 246119 | 0 – 60 cigarettes (ever-smoker) | 142202 |
| Current | 47640 |  |  |
| Covariates | | | |
| Gender | Count | Gender | Count |
| Female | 167329 | Female | 68425 |
| Male | 126430 | Male | 73777 |
| Genotype measurement batch | Count | Genotype measurement batch | Count |
| UK Biobank | 259621 | UK Biobank | 117276 |
| UK BiLEVEAX | 34138 | UK BiLEVEAX | 24926 |
| Total | 293759 | Total | 142202 |
| Age | Mean (SD) | Age | Mean (SD) |
| 55.89 ± 8.11 | | 57.58 ± 7.83 | |
| Body mass index | Mean (SD) | Body mass index | Mean (SD) |
| 27.11 ± 4.76 | | 27.9 ± 4.84 | |
| **Causal pathway discovery analysis** | | | |
| Smoking status (SS) | Count | Cigarette per day (CPD) | Count |
| Never | 21463 | 0 – 60 cigarettes (ever-smoker) | 8830 |
| Current | 2161 |  |  |
| Covariates | | | |
| Gender | Count | Gender | Count |
| Female | 12960 | Female | 4313 |
| Male | 10664 | Male | 4517 |
| Total | 23624 | Total | 8830 |
| Age | Mean (SD) | Age | Mean (SD) |
| 54.18 ± 7.39 | | 56.01 ± 7.26 | |

**Table S2**. White matter fractional anisotropy measures in UK Biobank by brain regions

| **Region of Interest** | **Hemisphere** | **Measure** | **Region of Interest** | **Hemisphere** | **Measure** |
| --- | --- | --- | --- | --- | --- |
| Inferior cerebellar peduncle | Whole | Fractional anisotropy | Posterior thalamic radiation | Left | Fractional anisotropy |
| Genu of corpus callosum | Whole | Fractional anisotropy | Posterior thalamic radiation | Right | Fractional anisotropy |
| Body of corpus callosum | Whole | Fractional anisotropy | [Sagittal stratum](http://biobank.ndph.ox.ac.uk/showcase/field.cgi?id=25086) | Left | Fractional anisotropy |
| Splenium of corpus callosum | Whole | Fractional anisotropy | [Sagittal stratum](http://biobank.ndph.ox.ac.uk/showcase/field.cgi?id=25086) | Right | Fractional anisotropy |
| Fornix | Whole | Fractional anisotropy | External capsule | Left | Fractional anisotropy |
| Corticospinal tract | Whole | Fractional anisotropy | External capsule | Right | Fractional anisotropy |
| Corticospinal tract | Left | Fractional anisotropy | [Cingulum cingulate gyrus](http://biobank.ndph.ox.ac.uk/showcase/field.cgi?id=25091) | Left | Fractional anisotropy |
| Corticospinal tract | Right | Fractional anisotropy | [Cingulum cingulate gyrus](http://biobank.ndph.ox.ac.uk/showcase/field.cgi?id=25091) | Right | Fractional anisotropy |
| Anterior limb of internal capsule | Left | Fractional anisotropy | [Cingulum hippocampus](http://biobank.ndph.ox.ac.uk/showcase/field.cgi?id=25093) | Left | Fractional anisotropy |
| Anterior limb of internal capsule | Right | Fractional anisotropy | [Cingulum hippocampus](http://biobank.ndph.ox.ac.uk/showcase/field.cgi?id=25093) | Right | Fractional anisotropy |
| Posterior limb of internal capsule | Left | Fractional anisotropy | Fornix cres+stria terminalis | Left | Fractional anisotropy |
| [Posterior limb of internal capsule](http://biobank.ndph.ox.ac.uk/showcase/field.cgi?id=25074) | Right | Fractional anisotropy | Fornix cres+stria terminalis | Right | Fractional anisotropy |
| [Retrolenticular part of internal capsule](http://biobank.ndph.ox.ac.uk/showcase/field.cgi?id=25076) | Left | Fractional anisotropy | Superior longitudinal fasciculus | Left | Fractional anisotropy |
| [Retrolenticular part of internal capsule](http://biobank.ndph.ox.ac.uk/showcase/field.cgi?id=25076) | Right | Fractional anisotropy | Superior longitudinal fasciculus | Right | Fractional anisotropy |
| Anterior corona radiata | Left | Fractional anisotropy | Superior fronto-occipital fasciculus | Left | Fractional anisotropy |
| Anterior corona radiata | Right | Fractional anisotropy | Superior fronto-occipital fasciculus | Right | Fractional anisotropy |
| [Superior corona radiata](http://biobank.ndph.ox.ac.uk/showcase/field.cgi?id=25080) | Left | Fractional anisotropy | Uncinate fasciculus | Left | Fractional anisotropy |
| [Superior corona radiata](http://biobank.ndph.ox.ac.uk/showcase/field.cgi?id=25080) | Right | Fractional anisotropy | Uncinate fasciculus | Right | Fractional anisotropy |
| [Posterior corona radiata](http://biobank.ndph.ox.ac.uk/showcase/field.cgi?id=25082) | Left | Fractional anisotropy | Tapetum | Left | Fractional anisotropy |
| [Posterior corona radiata](http://biobank.ndph.ox.ac.uk/showcase/field.cgi?id=25082) | Right | Fractional anisotropy | Tapetum | Right | Fractional anisotropy |

**Table S3**. Summary of numbers of FA significant negatively associated with respectively smoking-related phenotypes.

**Table S4.** Results of conditional independence test between each smoking-related phenotypes and FA measures given the genetic effects of each of the pleiotropic SNP.

**Table S5**. Functional annotations of the identified 22 pleiotropic variants associated with both CPD and regional FA measures (ALIC-R and PCR-L), and 272 variants associated with SS and ALIC-R from FAVOR.

**Table S6.** Summary of complete mediation analysis results from the causal pathway analysis between SNP, smoking-related phenotypes and FA measures.

**References**

Bogomolov M, Heller R. 2013. Discovering findings that replicate from a primary study of high dimension to a follow-up study. Journal of the American Statistical Association.108:1480-1492.

Hernán MA, Robins JM. 2010. Causal inference. In: CRC Boca Raton, FL;.

Imai K, Keele L, Yamamoto T. 2010. Identification, inference and sensitivity analysis for causal mediation effects. Statistical science.51-71.

Imbens GW, Rubin DB. 2015. Causal inference in statistics, social, and biomedical sciences: Cambridge University Press.

Sampson JN, Boca SM, Moore SC, Heller R. 2018. FWER and FDR control when testing multiple mediators. Bioinformatics.34:2418-2424.

Tingley D, Yamamoto T, Hirose K, Keele L, Imai K. 2014. Mediation: R package for causal mediation analysis. Journal of Statistical Software.

Tyler V. 2016. Mediation analysis: a practitioner's guide. Annual review of public health.37:17-32.
